# Land Use Intensification Effects in Soil Arthropod Community of an Entisol in Pernambuco State, Brazil

**DOI:** 10.1155/2014/625856

**Published:** 2014-10-20

**Authors:** G. M. Siqueira, E. F. F. Silva, J. Paz-Ferreiro

**Affiliations:** ^1^Center of Agricultural and Environmental Sciences, Federal University of Maranhão, BR-222, KM 04, Boa Vista, s/n, 65500-000 Chapadinha, MA, Brazil; ^2^Department of Rural Technology, Federal Rural University of Pernambuco, Dom Manoel de Medeiros, s/n, 52171-900 Recife, PE, Brazil; ^3^Faculty of Sciences, University of Coruña, Campus A Zapateira, 15008 Coruña, Spain

## Abstract

The interactions between soil invertebrates and land use and management are fundamental for soil quality assessment but remain largely unaddressed. The aim of this study was to evaluate the changes in soil arthropod community of an entisol brought about by different land use systems under semiarid climate in Pernambuco State, Brazil. The soil invertebrate community was sampled using pitfall traps from areas with eight vegetation types by the end of the austral winter. The land uses studied were native thorn forest plus seven agricultural fields planted with elephant grass, apple guava, passion fruit, carrot, maize, tomato, and green pepper. Native vegetation was considered as a reference, whereas the agricultural fields showed a range of soil use intensities. The abundance of organisms, the total and average richness, Shannon's diversity index, and the Pielou uniformity index were determined, and all of these were affected by several crop and soil management practices such as residue cover, weed control, and pesticide application. Our study found differences in community assemblages and composition under different land use systems, but no single taxa could be used as indicator of soil use intensity.

## 1. Introduction

Soil fauna include a large number of species that play a central role in many essential ecosystem processes [1, 2]. When a natural system is shifted by human activities for agricultural or forestry purposes, major changes occur in the soil environment and in the fauna populations and community. The intensity of the modifications induced by land use changes compared with the original ecosystem and the ability of the various soil organisms to adapt to these changes will determine the ultimate community present after the perturbation [3].

Agricultural practices can have a dramatic effect upon soil invertebrate community. Practices generally considered as beneficial for the soil fauna include the management of organic matter, particularly the control of the quality or quantity of plant residues and the absence of soil tillage. Also crop rotation, fertilization, and liming may also play an important role in increasing the diversity of soil biota. The main practices generally considered as having negative effects on soil biota comprise the use of pesticides, frequent and deep tillage, inadequate soil cover and poor management of organic residues, physical degradation, contamination, and pollution [3, 4].

There is a current interest in improving both soil quality and sustainable land management systems [5, 6]. Intensive land use can lead to negative impacts on soil quality. While physical and chemical properties have been demonstrated to respond slower to changes in soil use and management, it is widely accepted that soil biological and biochemical properties and also soil organisms are suitable indicators of soil quality [4–9].

Macrofaunal organisms have been widely accepted as indicators of soil quality. This is due to the important role of fauna regulating processes such as the formation and stability of soil aggregates, nutrient cycling, and soil aeration. Soil fauna provides top-down regulation of microbial responses to soil quality alterations via the regulation of the bacterial and fungal food webs in the soil. Other physical processes such as erosion and filtering can also be affected by soil fauna [1, 4, 7]. In addition, soil fauna measurements provide some advantages compared to other biological methods to measure soil quality as they rely on identifying and quantifying species living in the soil rather than on nonstandardized chemical analyses.

In particular, soil arthropods have been used as indicators of soil quality in soils and to compare different management systems, as they are regulated by anthropogenic impacts. Frequently, these studies have been done using single taxon groups, including Acari, Isopoda, Coleoptera, Araneae, or Collembola [8–10] or integrative quality indices [11, 12].

The interior region of northeastern Brazil is covered by xeric shrubland and thorn forest, locally referred to as “Caatinga,” unlike the Atlantic rain forest, which borders the Atlantic see. The “Caatinga” biome, located between 3°S 45°W and 17°S 35°W, incorporates about 900,000 km². This native vegetation typically consists of small, thorny trees; herbaceous vegetation only starts growing in the rainy season. Due to the high demographic density (20–30 inhabitants per km^2^) this is one of the most densely populated semiarid regions of the world. Therefore, there is a pressure to cultivate as much land as possible. Moreover, agricultural land use involves a large number of small properties with contrasting management systems, including irrigated, high input systems and rainfed, low input systems [13].

Intensive agricultural systems can produce negative impacts on soils, including a loss of soil quality and biodiversity. These aspects should be evaluated as soil fauna is sensitive to several soil management practices, including fertilizer use and tillage [14] or land use changes [15, 16]. Soil fauna is also sensitive to land degradation [17].

In spite of the essential role of arthropods in soil functioning, there is not enough knowledge about how they are affected by extensive and/or intensive agricultural systems. In particular, the arthropod community along intensification gradients has not been studied at the Brazilian Caatinga. Thus, in this work, we used soil arthropods as a mean to evaluate the effects of agricultural intensification on soil quality.

## 2. Material and Methods

### 2.1. Study Site, Climate, Soil Type, and Land Use

The study area was located in Fazenda Nossa Senhora do Rosário, Pesqueira municipality (Pernambuco, Brazil), at 8°34′17′′S and 37°1′20′′W. The average altitude is 610 m. The climate in the region is hot, semiarid, with dry austral summers and more rainy winters (BShw according to Köppen). Average annual temperature is 27°C and average annual rainfall is 600 mm. Usually, the rainy season starts in December or January.

**Figure 1:**
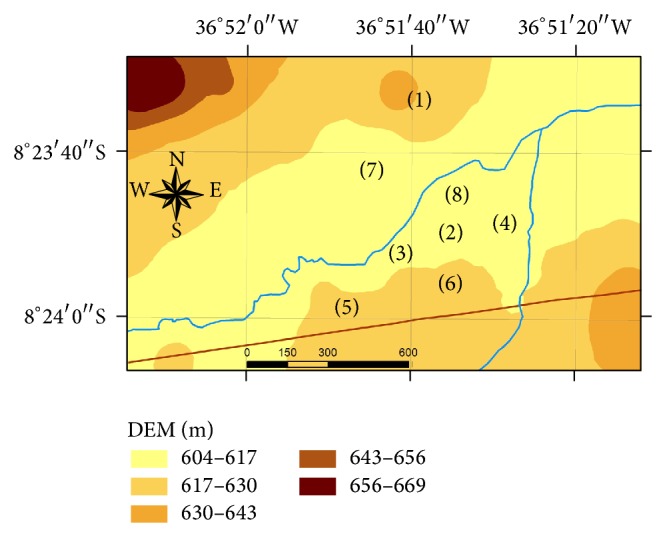
Localization of the studied stands (scale = meters).

Fazenda Nossa Senhora do Rosário is located on an alluvial valley (Figure 1). The soils of the studied fields were entisols, and they were classified as Fluvent and Orthent at the suborder level, following the Soil Survey Staff [18]. Fluvent and Orthent are equivalent to “Neossoo flúvico” and “Neossolo Regolítico,” respectively, in the Brazilian Soil Classification System [19]. General properties of these two soil types are listed in Tables 1 and 2. Both Fluvent and Orthent were sandy loam textured. Soil pH at the surface horizon (0–20 cm) was slightly acid, thus in the desired range for agricultural soils. Cation exchange capacity (CEC) was low, as expected for tropical soils, with values of 8,2 Cmol_+_ kg^−1^ and 6,6 Cmol_+_ kg^−1^ for Fluvent and Orthent, respectively. Organic matter content, however, was much higher for Orthent than for Fluvent.

In this area, production is carried out by small scale farmers, owning plots along the river valley. Some fields are used as forage for cattle, while most fields are subject to cultivation. The invertebrate community was evaluated in 8 plots, located within a thorn forest (which was taken as a reference) and in 7 agricultural fields with different cultivation systems and vegetation cover. Details about soil type, land use and soil and crop management of the sites studied are summarized in Table 3. A soil use intensity index for each of the studied stands was elaborated based on local expert knowledge and is also listed in Table 3.

The native forest (Caatinga) is a xeric or even hyperxeric vegetation with thorny tress as dominant species. In winter periods the small thorny trees lose their leaves and the understory merely consists of cacti, thick-stemmed plants, and arid-adapted grasses, limiting the litter available for soil biota. During the brief rainy season, however, many annual plants grow, flower, and produce much more abundant litter debris.

The agricultural fields studied were cropped with different plant species, including elephant grass (*Pennisetum purpureum*, Schumach), apple guava (*Psidium guajava*, L.), passion fruit (*Passiflora edulis, *Sims), carrot (*Daucus carota*, L.), maize (*Zea mays*, Mill.), tomato (*Solanum lycopersicum*, Mill.), and green pepper (*Capsicum annuum*, L.).

### 2.2. Sampling and Analysis of Active Fauna

Soil fauna was sampled using the pitfall trap method during a seven-day period, from 22 to 29 August, 2013. Average temperature during the sampling period was 21.5°C (average maxima 28.2°C and average minima 16.0°C). Due to high evaporation rates the soil was dry, even if two significant rainfall events were recorded before starting field fauna sampling; these events occurred on 1 August (20 mm) and on 19 August (10 mm). Pitfall traps were made of plastic (9 cm height × 8 cm diameter) [20]. Five traps were installed per land use. Each trap remained active for a week. The glass was set flush with the soil surface and contained 200 mL of formaldehyde (4%). The content of each glass was emptied afterwards in the field into a container, which was completed with 70% alcohol for preservation of the specimens.

Each sample was processed in the laboratory, separating and classifying the arthropods. All the adult and juvenile specimens were classified to the order level using a binocular microscope and taxonomic keys, with some exceptions. Within the order Hemiptera, the suborder Heteroptera was separated from the suborders Auchenorrhyncha and Sternorrhyncha. Acari was considered as an order. The total number of individuals were first counted per pitfall and taxa studied.

The data were also converted into number of individuals per trap per day. This parameter indicates the fauna abundance and was calculated for each taxonomic group and for the total arthropod community sampled. Aside from abundance, diversity of soil fauna was evaluated using the following ecological indices: mean richness (mean number of taxa trapped per pitfall and land use), total richness (total number of taxa trapped per land use, regardless of trap), Shannon diversity index and Pielou equitability (or evenness) index.

The Shannon-Weaner index was obtained as

(1) $$\begin{array}{c} H=-\sum pi\cdot \log2\cdot pi, \end{array}$$

where *pi*: *ni*/*N*; *ni* is number of individuals per trap per day for each of the orders studied (Ind*·*arm*·*dia^−1^), and *N* is sum of individuals per trap per day. This index describes the diversity as a weighted geometric mean of the relative distribution of abundance of the individuals between the groups sampled.

The Pielou evenness index is indicative of the uniformity of soil fauna for each land use and is calculated as follows:

(2) $$\begin{array}{c} U=\frac{H}{\log2S}, \end{array}$$

where *H* is Shannon index and *S* is remaining groups present in treatments.

This index quantifies how equal the community is numerically. Close relative abundance of the studied taxa is indicated by high values of Pielou index.

The Kolmogorov-Smirnov test was applied to assess the normality of the distribution of the data sets. Data were log-transformed to meet the requirements for parametric statistical tests. Only those arthropod groups that met the statistical assumptions were analyzed. Thus, the groups analyzed were available in high numbers. Moreover, most of the taxa were not assessed adequately with pitfall traps and were excluded from further analyses. As an example, flying organisms are trapped with different techniques depending on their behavior (e.g., stick and pheromone traps).

We used a one-way ANOVA to investigate the effects of land use intensification on the densities of soil surface-active arthropods. Differences between groups were assessed using the Tukey test.

## 3. Results and Discussion

All together, 6340 specimens were collected by pitfalls during the sampling period of seven days. They were grouper per land use and taxonomic group (Table 4). The total number of individuals trapped per land use showed wide differences. The largest arthropod community was found under apple guava (3224 individuals), but carrot (959 individuals) and green pepper (974 individuals) also showed much larger fauna recovery than other land uses. The smallest arthropod communities were collected under maize (59 individuals) and tomato (95 individuals). Under native forests 286 individuals were trapped, which is mostly comparable to elephant grass (330 individuals) and passion fruit (213 individuals). Entomobryomorpha was the dominant taxonomic group under apple guava (3080 individuals) and green pepper (780 individuals), whereas Poduromorpha prevailed under carrot (716 individuals).

Several factors may influence the soil invertebrate community, particularly season, microclimate, soil, and crop management, and resource availability [16, 17, 21]. With regard to the season, indeed rainfall might contribute to a more favorable environment for fauna activity at the semiarid region of northeast Brazil. In the studied area, however, water provided by irrigation has to be taken into account. Therefore, nonirrigated (elephant grass, apple guava, and passion fruit) and irrigated (passion fruit, carrot, maize, tomato, and green pepper) stands will show contrasting soil water content and microclimatic conditions that may influence arthropod density. Tillage system and soil cover are factor affecting the organic matter and litter production in agricultural fields and therefore the food availability for soil fauna. Thus, a higher litter production is expected under native forest, apple guava, and passion fruit, because plant residues are left on the soil surface; the more the litter production, the higher food availability for soil fauna.

Mainly two crop protection practices are expected to affect the soil fauna activity: weed control and pesticide application. At the sampling date weeds had been strictly excluded from passion fruit and tomato fields, because these crops were in the main fruiting phase. However, under apple guava, carrot, and green pepper the presence of weeds was not a challenge, because when sampling was performed, the crop production was out. As herbivores, Entomobryomorpha and Poduromorpha are enhanced by the presence of weeds. Thus, it is highly possible that the large number of Entomobryomorpha under apple guava and green pepper (followed by important figures for carrot) and also the dominance of Poduromorpha under carrot was related to the increased food availability, due to no restriction of weed growth.

Pesticides have been applied to carrot, maize, tomato, and green pepper. The low number of individuals collected under maize and tomato may be related to the use of pesticides together with the scarce or even absent soil cover and strict weed control. Carrot and green pepper also received pesticide treatment; however, these crops were near the end of the vegetative growth period and weeds provided food availability for certain specialized arthropod groups. Therefore, in these stands, Entomobryomorpha and Poduromorpha found a favorable environment, even after pesticide application.

The arthropods extracted from all the land uses belong to 18 taxa, with a minimum of 9 at carrot, maize, and tomato, and a maximum of 16 at passion fruit and the native forest (Table 5). The fauna richness therefore was lower in stands treated with pesticides (carrot, maize, tomato, and green pepper) than in stands without pesticide application (elephant grass, apple guava, and passion fruit) and in the native forest. The taxa exhibiting the greatest number of specimens were Entomobryomorpha, Poduromorpha, Formicidae, Diptera, Auchenorrhyncha, Araneae, Hymenoptera, and Thysanoptera, accounting for approximately 95% of the collected organisms.

In general, we found very strong effects of land use on the arthropod abundance. Several taxonomic groups showed higher abundance under specific crops. Entomobryomorpha were higher in plots under green pepper and apple guava (*F* = 4.58, *P* < 0.010). Poduromorpha were much higher in the plots under carrot than in any other treatment (*F* = 33.21, *P* < 0.001). Higher amounts of Auchenorrhyncha were found in the traps located in passion fruit, tomato, and green pepper plots (*F* = 15.20, *P* < 0.001). Thysanoptera was higher in soils under carrot and maize (*F* = 15.08, *P* < 0.001). Other taxa, like Coleoptera were ubiquitous; their abundance was similar in all the investigated land uses, but they were less abundant than other taxa. On the other hand, Formicidae were higher in the reference stand, under native vegetation, and in the stand under apple guava than in other cultivated stands (*F* = 7.01, *P* < 0.001). Also Hymenoptera were higher in the soil under native than in soils cropped with maize or tomato (*F* = 4.03, *P* < 0.010).

The pitfall trap is thought to be a sampling method most adequate for Araneae, Coleoptera, Formicidae, and Orthoptera, whereas other soil taxa should be investigated using other different methods [17]. The communities of Formicidae collected were significantly lower in all the cultivated stands than in the native forest stand, and this is irrespective of soil and crop management system. Therefore, this taxonomic group show promise as an indicator of soil quality in the Brazilian semiarid, because it exhibits the most pronounced decrease in cultivated plots compared to native forest. Assessment of this taxonomic group may be useful to evaluate the biological status of the cultivated stands.

The abundances of soil arthropods (individuals/pitfall/day) were lowest for maize (1.69 ± 0.51) and tomato (2.71 ± 0.49) and highest for apple guava (92.63 ± 64.42), followed by green pepper (27.83 ± 5.81) and passion fruit (27.40 ± 12.54), as shown in Table 5. Again, high pesticide application rates and weed control at sampling date were responsible for the small arthropod abundance under maize and tomato. On the other hand, the highest abundance of arthropods under apple guava and the relatively high abundance under passion fruit and green pepper was due to the effect of weed population, which dramatically increased the presence of specialized herbivores, as before mentioned.

Under native Caatinga, the abundance of arthropods was 8.17 ± 2.83 individuals/pitfall/day, intermediate in comparison with the various agricultural land uses. As expected, the fauna abundance under this biome was lower than in other tropical soils. For example, under secondary Atlantic forest the figures obtained using also pitfall traps ranged from 80.13 to 111.37 individuals/pitfall/day [21], depending on the sampling season, rainy or dry, respectively.

The Shannon index suggested that diversity was highest not only at the reference stand (native forest) but also at tomato and passion fruit; it was lowest at apple guava, followed by carrot and green pepper. Thus, this index ranked as passion fruit > tomato > native forest > maize > elephant grass > green pepper > carrot > apple guava. The Pielou index showed the highest evenness or equality at tomato and maize and the lowest at apple guava. It ranked as tomato > maize > passion fruit > native forest > green pepper > carrot > apple guava. The arthropod communities under tomato and maize exhibited a high diversity and evenness (high Shannon and Pielou indices), and this is in spite of pesticide application and weed control. Therefore, such crop management practices affected fauna abundance, but not biodiversity and evenness. On the other hand, the presence of weeds dramatically increased the abundance of some taxa and this lead to a lower diversity and a lower equitability under apple guava, carrot, and green pepper.

Surprisingly, agricultural intensification does not seem to cause always a decrease of arthropod diversity and evenness. Moreover, high taxa richness could be a consequence of a shift in community composition towards a high number of taxa better adapted to the conditions in these soils. It should be also taken into account that diversity indices are known to be sensitive to various factors, such as the sampling unit size which limits their predictive capability [22].

The soils under different land uses widely differed in invertebrate density, whereas they differed more slightly in taxa richness. Therefore, we can hypothesize that individual abundance and density are affected by different soil management factors than taxa richness. It appeared that in agricultural land uses abundance was mainly related to the presence of weeds, while richness was associated to pesticide application. This is a result consistent with previous studies [22, 23].

On the other hand, the soil use intensity index (Table 5) showed no significant correlation with the studied biodiversity indices or with single taxa abundance. This may be simple due to the semiquantitative nature of the soil use intensity index.

The community composition did greatly differ among the studied land uses, which would have severe implications for soil functionality [23]. As an example, several arthropods, including mites and termites, are involved in organic matter decomposition and nutrient cycling. In addition, ants and other arthropods create channels, aggregates, and mounds that deeply affect the fluxes of gases and water in soil. This physical alteration will also modify the microhabitats for other soil organisms.

The high relative abundance of Auchenorrhyncha, Entomobryomorpha, Hymenoptera, Poduromorpha, Formiciadae, and Thysanoptera suggests that these taxa are tolerant to a wide range of soil properties. This would limit their use as indicators of soil conditions and, thus, soil quality [23]. These taxa, however, showed variations in individual abundance among the different management systems, which may be due to high food availability when sampling. As an example, Entomobryomorpha had a 100-fold higher abundance in the soil under apple guava than in the soils under tomato or maize. Again Formicidae among the ubiquitous taxa appears to be the most sensitive to changes in land use and soil management.

Other taxa such as Acari, Araneae, Auchenorrhyncha, Hymenoptera, Isoptera, Orthoptera, Poduromorpha, and Sternorrhyncha were absent in some treatments, which may suggest a good potential as indicators of soil quality. In particular, Acari was absent under maize and almost absent under tomato and green pepper, while Poduromorpha were absent under maize and green pepper. This would indicate that these taxa are very sensitive to specific practices of intensification in soil and crop management. This is supported by the fact that Acari numbers and traits may change due to an intensification of soil use [24].

Overall, the highly variable density of microarthropods in this agricultural landscape seems to be dependent on several variables. Appropriate statistical analyses are required to identify those variables. Further research could be useful to better assess the sensitivity and the role of single species, since many species belonging to the same taxa and exhibiting different traits can adopt different strategies in the landscape. It is important to highlight that our study demonstrates there are differences in invertebrate communities between land uses, even at taxa levels, that can be attributed to intensive agricultural management. This leads to significant differences that are indicated by the presence or absence of arthropod taxa.

## 4. Conclusions

The abundance of arthropods under native forest in the Brazilian semiarid has been found to be much lower than under other native biomes, such as the Atlantic forest.

Under agricultural land use several variables related with soil and crop management influence the highly variable density of arthropods. Abundance has been shown to be related with food availability provided by weeds, which dramatically increased the presence of specialized herbivores, such as Entomobryomorpha and Poduromorpha. Richness was mainly associated with pesticide application.

Agricultural land use strongly decreased the abundance of Formicidae compared to native forest, which suggest this taxonomic group responds sensitively to agricultural land use. Several other taxonomic groups showed significantly higher abundances under specific crops, but this effect may be due to distinct soil and crop management practices at the sampling date. However, no single taxa could be used as indicator of soil use intensity.

## Figures and Tables

**Table 1:** Granulometric analysis as a function of depth of the soils studied.

Fluvent (Neossolo Flúvico)				Orthent (Neossolo Regolítico)			
Depth (cm)	Sand (%)	Silt (%)	Clay (%)	Depth (cm)	Sand (%)	Silt (%)	Clay (%)
0–20	64.69	19.78	15.44	0–15	65.68	18.00	16.32
20–40	64.29	18.41	17.30	15–35	60.24	20.56	19.20
40–60	68.39	15.65	15.97	35–70	63.52	15.28	21.20

**Table 2:** General chemical properties of the soils studied.

Depth (cm)	Fluvent (Neossolo Flúvico)									
	pH	P	Na	K	Mg	Ca	H	CEC	OC	OM
	H_2_O	mg/dm^3^	cmolc/dm^3^						g/kg	
0–20	6.2	100	0.14	0.25	1.7	2.8	3.31	8.20	0.24	0.42

Depth (cm)	Orthent (Neossolo Regolítico)									
	pH	P	Na	K	Mg	Ca	H	CEC	CO	MO
	H_2_O	mg/dm^3^	cmolc/dm^3^						g/kg	

0–20	6.5	41	0.09	0.28	0.85	1.85	3.51	6.58	4.36	7.52

(CEC = cation exchange capacity; OC = organic carbon; OM = organic matter).

**Table 3:** Location, soil type, and soil and crop management of the studied natural and agricultural stands.

Coordinates	Altitude	Soil type (SSA)	Soil type (BSCS)	Vegetation	Tillage and soil cover	Weeds at sampling	Irrigation	Pesticides	Soil use intensity
8°23′38.84′′S 36°51′34.52′′W	634 m	Fluvent	Neossolo Flúvico	Natural vegetation (*Caatinga Biome*)	Uncultivated, soil 100% covered	—	No	No	0

8°23′47.26′′S 36°51′35.56′′W	611 m	Fluvent	Neossolo Flúvico	Elephant grass (*Pennisetum purpureum*)	Established 2 years ago, periodic grass mow	—	No	No	1

8°23′52.93′′S 36°51′39.19′′W	612 m	Fluvent	Neossolo Flúvico	Apple guava (*Psidium guajava*)	Established 3 years ago, soil 100% covered	Yes	No	No	1

8°23′46.59′′S 36°51′33.15′′W	613 m	Fluvent	Neossolo Flúvico	Passion fruit (*Passiflora edulis*)	Six months fallow, between lines coverage	No	Drip	No	2

8°23′58.23′′S 36°51′39.06′′W	621 m	Orthent	Neossolo Regolítico	*Carrot* (*Daucus carota*)	Plowing and disk harrow, soil partially covered	Yes	Drip	Yes	3

8°23′54.43′′S 36°51′37.35′′W	615 m	Orthent	Neossolo Regolítico	*Maize (Zea mays) *	Plowing and disk harrow, soil partially covered	No	Aspersion	Yes	3.5

8°23′41.19′′S 36°51′37.54′′W	610 m	Fluvent	Neossolo Flúvico	Tomato (*Solanum lycopersicum*)	Plowing and disk harrow, uncovered between lines	No	Drip	Yes	5

8°23′45.61′′S 36°51′35.89′′W	610 m	Fluvent	Neossolo Flúvico	Green Pepper (*Capsicum annuum*)	Plowing and disk harrow, uncovered between lines	Yes	Drip	Yes	5

(SSA = Soil Survey Staff; BSCS = Brazilian Soil Classification System).

**Table 4:** Total number of individuals collected by 5 pitfall traps during a week for the taxonomic groups studied.

Taxonomic group	Native forest	Elephant grass	Apple guava	Passion fruit	Carrot	Maize	Tomato	Green pepper
Acari	8	5	18	2	3		1	1
Araneae	19	5	13	1		3		17
Auchenorryncha	1	4	2	24		1	25	43
Coleóptera	18	9	6	13	5	11	6	11
Diplura				1				
Diptera	51	18	22	38	9	13	13	24
Entomobryomorpha	67	251	3080	53	188	21	21	780
Formicidae	90	14	70	29	19	4	7	36
Heteroptera			1					
Hymenoptera	19	6	10	11			15	2
Isoptera	1	3	13		1			
Larva Coleoptera	1			1				4
Orthoptera	1	2	3	4		3		
Poduromorpha	2	1	2	1	716		4	
Psocoptera				2				
Sternorryncha	5	8		1	5	1		5
Thysanoptera	3	4	1	32	13	2	3	51
Tricoptera			1					
Total	**286**	**330**	**3424**	**213**	**959**	**59**	**95**	**974**

**Table 5:** Parameters and indices used to assess arthropod communities under native forest and the different agricultural land uses studied.

	Abundance ± std (Ind*·*pitfall*·*day^−1^)	CV	Shannon index	Pielou index	Mean richness	Total richness
Native forest	8.17 ± 2.83	34.7	2.709	0.712	8.4	14
Elephant grass	9.43 ± 3.56	37.8	1.641	0.444	8.4	13
Apple guava	92.63 ± 64.42	69.5	0.439	0.115	8.2	14
Passion fruit	6.09 ± 0.91	15.0	2.983	0.764	8.6	15
Carrot	27.40 ± 12.54	45.8	1.150	0.363	6	9
Maize	1.69 ± 0.51	30.2	2.562	0.808	4.4	9
Tomato	2.71 ± 0.49	18.2	2.749	0.867	7	9
Green pepper	27.83 ± 5.81	20.9	1.267	0.366	8	11
